# In Silico Mathematical Modelling for Glioblastoma: A Critical Review and a Patient-Specific Case

**DOI:** 10.3390/jcm10102169

**Published:** 2021-05-17

**Authors:** Jacopo Falco, Abramo Agosti, Ignazio G. Vetrano, Alberto Bizzi, Francesco Restelli, Morgan Broggi, Marco Schiariti, Francesco DiMeco, Paolo Ferroli, Pasquale Ciarletta, Francesco Acerbi

**Affiliations:** 1 Department of Neurosurgery, Fondazione IRCCS Istituto Neurologico Carlo Besta, 20133 Milan, Italy; jacopofalco910@gmail.com (J.F.); ignazio.vetrano@istituto-besta.it (I.G.V.); francesco.restelli91@gmail.com (F.R.); morganbroggi@hotmail.com (M.B.); marco.schiariti@istituto-besta.it (M.S.); francesco.dimeco@istituto-besta.it (F.D.); paolo.ferroli@istituto-besta.it (P.F.); 2 MOX, Department of Mathematics, Politecnico di Milano, 20133 Milan, Italy; abramoago@gmail.com (A.A.); pasquale.ciarletta@polimi.it (P.C.); 3 Department of Neuroradiology, Fondazione IRCCS Istituto Neurologico Carlo Besta, 20133 Milan, Italy; alberto.bizzi@istituto-besta.it; 4 Department of Pathophysiology and Transplantation, Università degli Studi di Milano, 20122 Milan, Italy; 5 Department of Neurological Surgery, Johns Hopkins Medical School, Baltimora, MD 21205, USA

**Keywords:** biomathematics, cancer modelling, diffusion tensor imaging, glioblastoma, in silico, personalized neuro-oncology

## Abstract

Glioblastoma extensively infiltrates the brain; despite surgery and aggressive therapies, the prognosis is poor. A multidisciplinary approach combining mathematical, clinical and radiological data has the potential to foster our understanding of glioblastoma evolution in every single patient, with the aim of tailoring therapeutic weapons. In particular, the ultimate goal of biomathematics for cancer is the identification of the most suitable theoretical models and simulation tools, both to describe the biological complexity of carcinogenesis and to predict tumor evolution. In this report, we describe the results of a critical review about different mathematical models in neuro-oncology with their clinical implications. A comprehensive literature search and review for English-language articles concerning mathematical modelling in glioblastoma has been conducted. The review explored the different proposed models, classifying them and indicating the significative advances of each one. Furthermore, we present a specific case of a glioblastoma patient in which our recently proposed innovative mechanical model has been applied. The results of the mathematical models have the potential to provide a relevant benefit for clinicians and, more importantly, they might drive progress towards improving tumor control and patient’s prognosis. Further prospective comparative trials, however, are still necessary to prove the impact of mathematical neuro-oncology in clinical practice.

## 1. Introduction

Glioblastoma (GBM) is the most common malignant primary brain tumor [1,2]. Its most important characteristics relies on the capability to extensively infiltrate the brain parenchyma at distance from the main tumor mass, usually following white matter tracts [3,4,5]. Despite aggressive multimodal therapy such as surgical resection, radiotherapy (RT) and chemotherapy (CHT), the overall median progression-free and overall survival times are approximately 7 and 15 months, respectively [6,7,8,9,10]. GBM, in fact, invariably recurs, usually growing at the margin of the surgical cavity, where the tumor has been resected [11]. However, the characteristics of tumor growth and recurrence are highly variable, and unpredictable [12]. Although the strict relationship between the tumor and brain eloquent areas sometimes plays a key role in limiting the development of aggressive local therapy to avoid the risk of inducing irreversible neurological deficits (i.e., oncofunctional balance), the highly variable pattern of tumor relapse complicates efforts to develop local chemotherapies and tailor surgical resection. Furthermore, at the moment the therapeutic options, especially radiotherapeutic regimes, are mostly standardized in every patient, without taking into account the specific growth characteristics of the tumor and the probability of invasiveness into the peritumoral area [13].

During the past few decades, studying the evolution of gliomas from a mathematical point of view has become an issue of primary importance, mainly because, despite the impressive scientific, medical and technological advances, a cure for this disease has not yet been found [14]. Therefore, numerous mathematical models have been elaborate to study and predict progression of tumor [15]. The aim of mathematical oncology is to standardize and categorize a world of complexity in order to apply a large amount of biological and clinical data [16]. In silico models simulate the behavior of a tumor, consisting of interacting components thanks to experimental information and clinical data [17]. Likewise, in silico models need to be continuously validated by in vitro or in vivo experiments to achieve a further optimization.

The evolution of cancer is a very complex phenomenon, in which key events occur on many different scales: subcellular (gene expression, transduction of chemical signals, regulation of receptors), cellular (interactions among tumor cells and other types, motility processes) and tissue (cell migration, diffusion of nutrients, cellular interactions with matrix and external tissues) levels [18]. Through the years, mathematicians have developed increasingly sophisticated models, in order to investigate several tumor evolution aspects and scenarios, such as genetic mutations, proliferation, invasion and spread, angiogenesis or therapies effects [19]. In particular, regarding mathematical models in neuro-oncology, in silico studies allowed for investigation and comparison of different radiation regimens as well as study of antiangiogenic factors in order to minimize patient suffering while maximizing treatment effectiveness [20]; indeed, RT is often underrepresented in clinical trials, which are mainly funded by the industry and focused on innovative drugs [21,22].

The aim of the present study is to present a comprehensive review on different mathematical models in neuro-oncology with several clinical implications. Furthermore, in this paper, we present a preliminary clinical case in which we have applied our innovative approach [23,24]: in particular, we recently developed a patient-specific mechanical model of GBM growth, taking into account biochemical and mechanical interactions between to tumor and its local microenvironment [25]. Based on this preliminary experience, we started a prospective observational trial, named GLIOMATH (GLIOblastoma MATHematics), enrolling patient with GBM submitted to surgical removal or biopsy, adjuvant therapy and follow-up based on normal clinical practice, in which specific MRI data for each patient were used as input data building a personalized virtual environment.

## 2. Materials and Methods

**Figure 1:**
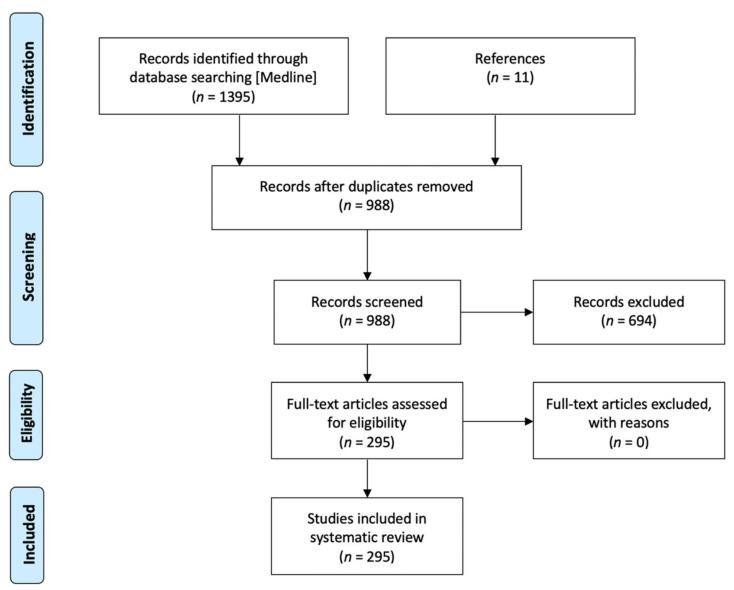
Flow chart of the inclusion process based on the “PRISMA 2009 flow diagram”.

A literature search regarding mathematical modelling for glioblastoma was performed in PubMed (Medline), according to the Preferred Reporting Items for Systematic Reviews and Meta-Analyses (PRISMA [26]) Statement. Studies published in the English language from 1 January 2001 to 31 December 2020, and for which at least the abstracts written in English with all necessary data were available, were considered for initial review. First of all, we used sequential keywords (mathematical neuro-oncology, mathematical glioma, in silico glioblastoma), and then checked for other possibly relevant articles in the references of the papers included in the analysis. We included clinical studies, clinical trials, observational studies and reviews with new modelling approach. We chose to exclude letters to the editor, pure reviews without new surgical records, case reports, surveys, editorials and clinical images. Excluding 418 duplicated articles, 977 articles were initially screened; 694 studied were excluded based on the inclusion and exclusion criteria of this review. In addition, 11 other articles were identified from the analysis of the references included in all the previous articles. Finally, as a result of our systematic analysis, 295 articles were considered (Figure 1).

Our review has been restricted to the innovative steps of in silico mathematical modelling to study glioblastoma growth, dedifferentiation and spread with different clinical applications.

Furthermore, in Appendix A and Appendix B we present the experimental details of our clinical trial and explain our model derivation.

## 3. Results

Mathematical oncology models of in vivo tumors can be divided into two broad categories depending on the scale at which the tumor is represented [27]: discrete or stochastic models focus on the microscopic scale, emphasizing the interplay at the cellular level; conversely, continuum or analytical models concern the macroscale, without being interested in what happens at subcellular level.

### 3.1. Continuum Models

Continuum models are simpler than discrete one, manage the glioma as a collection of tissue taking into account few parameters and describing it through cell densities, volume fractions and substrates concentrations; these models can accurately describe actual tumors and their response to treatment (Table 1).

The first mathematical models developed in tumor research followed a macroscopic approach and were called static, because they were based on the assumption that cancerous cells divide at rates, varying among different types of tumors, that could be considered approximately constant on large temporal scales [39]. The tumor growth was thereby described by an exponential law, modelling just cellular proliferation and neglecting every aspect connected to invasion [21].

After the pioneering work of Steel [28], who first analyzed the relation between cell kinetics and growth of the gross tumor, Murray’s group defined the basic spatio-temporal model for cancer evolution, which still followed an exponential law for cells duplication, but also took cellular diffusion into account [29,40]. This work is the basis of the most widely used analytical model, the proliferation—invasion (PI) model, upon which many others have been developed [41].

Subsequently, Swanson’s group [5] and Jbabdi’s group [30] reformulated the model using the recent results of brain MRI: the former incorporated the effects of augmented cell motility in white matter as compared to grey matter, quantifying the observation that glioma cells migrate more quickly in white matter. The latter further generalized this concept taking into account not only the heterogeneity of the brain tissue but also its anisotropy, dealing with the fact that glioma cell migration is facilitated in the direction of white matter fibers: they introduced the diffusion tensor obtained through the Diffusion Tensor Imaging (DTI) technique.

Cristini et al. [31] firstly identified the concept of tumor “diffusional instability” in a low vascularization setting as a mechanism for invasion: instability during growth can allow cancers to grow indefinitely, bypassing the symmetric steady state. Interestingly, the shape of highly vascularized tumors was predicted to remain compact and without invasive fingering, even while growing unboundedly; this suggested that the invasive growth and spread of malignant tumors is strictly correlated to vascular anisotropy in microenvironment. They simulated a continuum model of tumor growth and studied avascular and vascularized evolution in the nonlinear regime analyzing parameters involved in tumor morphology and growth on the basis of degree of vascularization. Indeed, the morphological evolution of a malignant glioma is the result of many factors, including intercellular adhesion, cell motility and interaction with extracellular matrix. In addition, the spatial and temporal distributions of oxygen and vital nutrients play an important role. Understanding the morphological stability of a cancerous tumor may be important for controlling its spread to surrounding tissue. Moreover, mathematical modelling shows that parameters controlling the tumor mass shape also rule its ability to invade, hence tumor morphology may serve as a predictor of invasiveness and treatment prognosis [17].

Lowengrub’s group [32,42] extended the previous work introducing a three-dimensions geometry; they confirmed morphologic instability showing how the tumor grows to larger sizes than would be possible if it were spherical. Furthermore, they confirmed that cancer morphology critically depends upon the properties of the microenvironment (presence of nutrients, easiness of mobility, different resistance of the extracellular matrix). These results suggest that development of malignant gliomas should be due to anisotropies in the distribution of the nutrient, of the mechanical resistance of the external tissue (grey, white matter and cerebrospinal fluid [CSF]) or of blood vessels.

Harpold et al. [21] derived the Fisher approximation introducing the velocity of the detectable tumor margin that is proportional to the proliferation rate of glioma cells and their diffusion coefficient in the health brain. Upon the basis of this approximation there was the observation that a cell population expands linearly in case of a standardized growth and diffusion alone.

Again, Swanson’s group [20] and Wang’s group [33,41] tested the concept of tumor spreading velocity starting from patient-specific MRI data; this last provided useful feedback in estimating diffusion rate for each patient. A personalized diffusion rate was derived from diagnostic T1 with paramagnetic contrast administration and T2 sequences tumor volumes. Furthermore, they tested their models in newly diagnosed GBM patients with the aim of predicting tumor behavior, patient survival and glioma response to adjuvant therapies.

Indeed, in the same years, Rockne et al. [34,43] incorporated in the mathematical model of glioma growth the effect of radiation therapy to produce a straightforward extension of current available technique. They simulated this model in a patient-specific series at the time of GBM diagnosis estimating, upon the basis of preoperative MRI, extent of resection (EOR) and RT protocol (fractioning, radiation dose and plan), both diffusion coefficient and proliferation rate with high accuracy and overlap between simulated and actual tumor post RT.

Subsequent research was aimed at improvement of the above model by introducing personalized coefficients and anisotropy on the proportion of white matter [44,45]; furthermore, malignant glial cells infiltration was taken into account in Unkelbach’s group model estimating this parameter from FLAIR images [35]. In particular [46], tumor cell density and diffusion rate were derived by patient-specific MRI.

Recently, research on GBM has focused on hypoxia as an indication of tumor’s aggressiveness and on angiogenesis, a hallmark of GBM [36]. A strong correlation was found between hypoxia and cellular diffusion coefficient and proliferation rate of the PI model; incorporating the effects of hypoxia, necrosis and angiogenesis, this new model has come to investigate the dynamics between glioma cells and their microenvironment, identifying predictable patterns of malignant progression, extent of hypoxic burden and size of the necrotic region in GBM.

Saut et al. [37] further developed a new grow-or-go multilayer model for GBM to address the link between hypoxia and tumor’s invasiveness. The grow-or-go theory hypothesizes that malignant glial cells switch from a proliferative to an invasive phenotype, mutually exclusive, on the basis of nutrients and oxygen provided by the tumor microenvironment.

The last, most significant innovation in mathematical modelling was the switching from the single-phase models to multiphase models that consider the tumor as composed by multiple phases including a variety of different cell genotypes and phenotypes as well as extracellular matrix and water [47,48]; however, tumor microenvironment and impaired glial genetic mechanisms can lead to multiple cell genotypes and phenotypes that select for glioma cell survival under abnormal conditions, with profound consequences on the overall GBM growth, invasion and response to treatment. Therefore, continuous multiphase models seem more suitable to precisely describe GBM growth process at a macroscopic scale, since they are able to incorporate the mass, momentum and energy balances that drive the carcinogenesis. In recent years, Lipkova and coworkers [38] integrated in a multiphase model the most relevant information derivable from both MRI and FET-PET to infer tumor cellular density in GBM patients; by means of this tool, the authors proposed how to tailor radiotherapy administration extending the capability of personalized schedule.

The model developed and proposed by our group [23,24] is a novel continuous multiphase model that successfully incorporates information from patient-specific DTI data: it takes into account patient-specific structural heterogeneity and anisotropy of the brain bundles. The model considers the viscous interactions among the phases and the mechanical interactions responsible of cell–cell and cell-matrix adhesion forces [49]; furthermore, it takes into account the directed motion of GBM cells towards increasing gradient of nutrients and along fibers path, as well as the preferential diffusion of blood, which means both nutrients and oxygen, and their gradient of concentration [5]. Particularly, our model combines the fundamental works achieved by Swanson’s group regarding the role of MRI in detecting brain white matter structure and by Cristini’s group concerning tumor invasion during its pathological growth.

### 3.2. Discrete and Hybrid Models

Discrete models are better equipped to study intracellular phenomena and emphasizes the interplay at the cellular level: individual cells are tracked and updated according to a specific set of biophysical rules (Table 2). This approach is particularly useful for studying carcinogenesis, genetic instability, immunological properties and response to molecular therapy [50,51].

The first discrete models developed were defined lattice-based because the cells were confined to a regular grid lattice in which each computational point is accommodated by a finite number of cells in specific states, derived from physical conservation laws and biological constraints. Subsequent research has overcome this model towards a lattice-free one (agent-based models) [63]; indeed, they allow more complex and accurate coupling between the cells and their microenvironment and imposes fewer artificial constraints on the behavior of multicellular systems. The cells are treated as distinct objects or agents and are allowed to move, divide and die individually according to biophysically based rules. Furthermore, the level of detail of the agents can be tailored to the simulation.

The bases of discrete models were founded by Duchting’s [52] and Wasserman’s [53] groups: the former developed a 3D model of a small tumor spheroid, focusing on tumor cell cycle phases and intercellular communication, whereas the latter highlighted mechanical properties of the tumor, implementing the model with patient-specific MRI.

Kansal et al. [54,55] applied a specific lattice-based model, defined cellular automaton, in which each site in the grid is accommodated by a finite number of cells in specific states; they described untreated GBM focusing on tumor dimension, probability of division and replicating fraction.

Stamatakos’ group [56] provided the most important innovations in the direction of clinical implications by attempting to connect cellular information to tissue level setting. They improved computational simulation performance and virtual reality. They integrated in a single model several groups of cells, both in the different phases of the cell cycle and outside the cycle (apoptosis and necrosis), to study different activities of each subpopulation of GBM. In particular, they derived these data from functional-metabolic imaging such as PET or BOLD-MRI. This innovation allows to study different protocol of RT administration, analyzing which malignant glial cells could be more radiosensitive and which therapeutic plan could have the best clinical response. Furthermore [64], since the discrete models emphasized cellular and subcellular scales, they were able to study different glioblastoma population upon different genetic profile and mutations.

Finally, Stamatakos et al. [57] refined their model taking into account the variations of radiosensitivity of biological cells according to the phase class, incorporating genetic and molecular factors affecting radiosensitivity. They continuously improve their model thanks to the simulation with real case of GBM patients to promote patient-specific treatments; they integrate personalized information from the molecular, histological and imaging level in order to simulate GBM growth and response to adjuvant therapies.

Hybrid modelling techniques, such as the latest innovations of Stamatakos’ group, have been proposed in the latest years with the aim of combining the advantages of the two main frameworks, continuum and discrete, in order to simulate multiscale problems and bridge the subcellular and cellular scales to the tumor scale. Hybrid models [59] may provide more realistic descriptions of microscopic mechanisms while efficiently evolving the entire system to obtain macroscopic observations [65].

Zheng et al. [58] extended the continuum model developed by Cristini and Lowengrub to include angiogenesis and an extratumoral microenvironment. The model was used to examine the competition between heterogeneous cell proliferation, caused by spatial diffusion gradients, and stabilizing mechanical forces and to predict changes in tumor morphology in response to perturbations in the model parameters that govern intercellular adhesion and the density of the microvasculature in the host tissue [66]; upon the basis of intercellular forces and angiogenesis grade, it is possible to predict GBM morphology and spread after surgical resection as well as to study innovative therapies such as anti-angiogenic factors.

Subsequently, Frieboes et al. [59] performed the first computational simulations of growing glioma and neovascular morphologies, finding superimposable results, that is that microvascular inadequacy may induce necrosis, drive glioma cells to aggressively invade adjacent tissue in vivo and trigger angiogenic sprouting from the surrounding microvasculature.

Recently, the proposed hybrid models focused on the pattern of malignant cell invasion as derivable from the combination of molecular cellular data and interactions with microenvironment. In particular, Kim and Roh [60] introduced a hybrid model that deepens the key mechanism behind the molecular switched between proliferative and migratory phase in response to the combination of metabolic stress and biophysical interactions of glioma spheroids with microenvironment with the aim of detecting biomolecular features accountable of tumor cell behavior and developing innovative, targeted therapies.

Angeli et al. [61] incorporated tumor biomechanical response at tissue level taking into account cellular events that can modify cancer proliferation, infiltration and invasion combining MRI information (DTI and PWI) with molecular subcellular data: the proposed mathematical framework aims to study tumor infiltration and distant invasion. Subsequently, Gallaher’s group [62] studied the tissue-scale dynamics (i.e., *microenvironment*) with serial MRI acquisition and combined these periodic data with molecular information as detectable from tumor biopsy; the mathematical framework proposed by the authors allowed to study tumor infiltration and migration and the possibility of response to different therapeutic approaches. 

### 3.3. Clinical Case Study: Primary GBM Tumor

**Figure 2:**
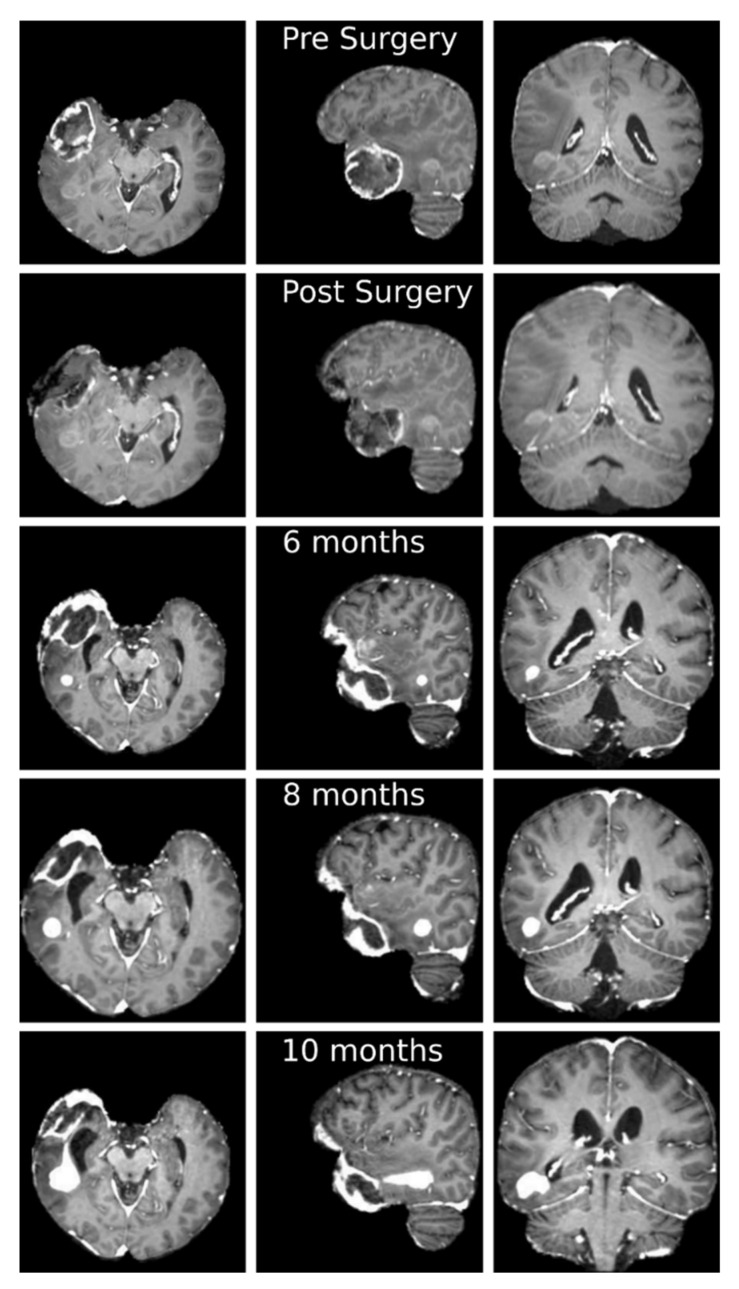
Axial (first column), sagittal (second column) and coronal (third column) slices of the T1-weigthed post contrast administration MRI at different temporal stages. First row: before surgery; second row: after surgery; third row: 6 months after surgery; fourth row: 8 months after surgery; fifth row: 10 months after surgery. It is possible to appreciate the gross total resection of the temporal pole lesion and a progressive volumetric increase of the posterior temporal mass, with change in contrast enhancement characteristics, from the sixth month after surgery.

Clinical trial and mathematical model derivation are described in Appendix A and Appendix B; we present, as exemplificative case, a 55-year-old patient with a right temporal lesion. The preoperative MRI according to our protocol documented the presence of a 4.9 × 4.3 × 3.6 cm mass in the right temporal pole, with inhomogeneous, peripheral contrast enhancement and a necrotic core, suggestive for high-grade glioma; a sub-centimetric posterior temporal lesion with moderate contrast enhancement was visible too (Figure 2). He underwent complete removal of the voluminous contrast enhancing lesion without any neurological worsening. Histopathological analysis confirmed the suspected diagnosis of *glioblastoma* (WHO IV) and methylation status of MGMT gene promoter.

The patient started RT with concomitant CHT (temozolomide) according to Stupp protocol 25 days after surgery; the pre-RT MRI confirmed, together with the surgical cavity due to tumor removal, the presence of a posterior temporal lesion that was considered in the simulation as an independent tumor. The patient completed the standard radiation protocol (60Gy in 30 fractions) with concomitant and adjuvant (6 cycles) temozolomide. The patient performed post-RT MRI immediately after the treatment and then every two months. These MRI showed the progression of the posterior temporal lesion, until a clear tumor nodule was evident 10 months after the first surgery (4.2 × 2.9 × 2.7 cm). For this reason, the patient underwent a second surgical procedure, with a total removal of the lesion, resulted to be again a *glioblastoma* (WHO IV) and non-methylation status of MGMT gene promoter. Mathematical simulation in this case was performed considering the posterior lesion as a separate location compared to the main tumor mass, followed-up until clear tumor volume increasement.

Before surgery we could observe a voluminous mass in the right temporal pole and a sub-centimetric posterior lesion (Figure 2). In particular, the latter one began to grow 6 months after surgery with an increase and a change in the contrast enhancement characteristics. During the two subsequent months, the small tumor mass grew in a regular manner following the anisotropic structure of the underlying tissues. In the last two months, before the second surgery, the tumor mass grew in an irregular branching manner, connecting to the regions infiltrated by the removed tumor mass.

**Figure 3:**
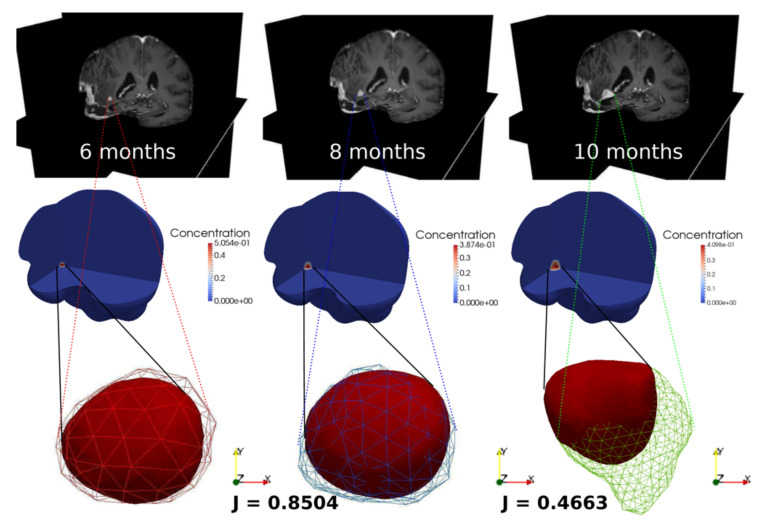
Simulated GBM evolution (**bottom**) against the MRI images (**top**) at 6 months, 8 months and 10 months after surgery. J is the Jaccard index, the color bars refer to the GBM volume fraction.

The resulting simulation depicts the tumor evolution from 6 months to 10 months after surgery, together with the corresponding MRI images (Figure 3). We can observe that the tumor contours from the simulation and the data almost overlap at 8 months. Indeed, we have selected a set of values for the growth parameters in the model in such a way that the volume values from the simulation is matching at 99% the volume value from the data. We have also computed the Jaccard index, which measures the overall overlap between the tumor extensions from simulation and MRI data, which is equal to 0.8504 at 8 months, showing a very high matching compared to results obtained in the literature. At the 10 months stage, the simulation results are not able to catch the real tumor dynamics in which the tumor is connecting to the infiltrated left particles from the removed tumor during surgery and is following the high chemotactic gradient towards the region filled by CSF after surgery.

**Figure 4:**
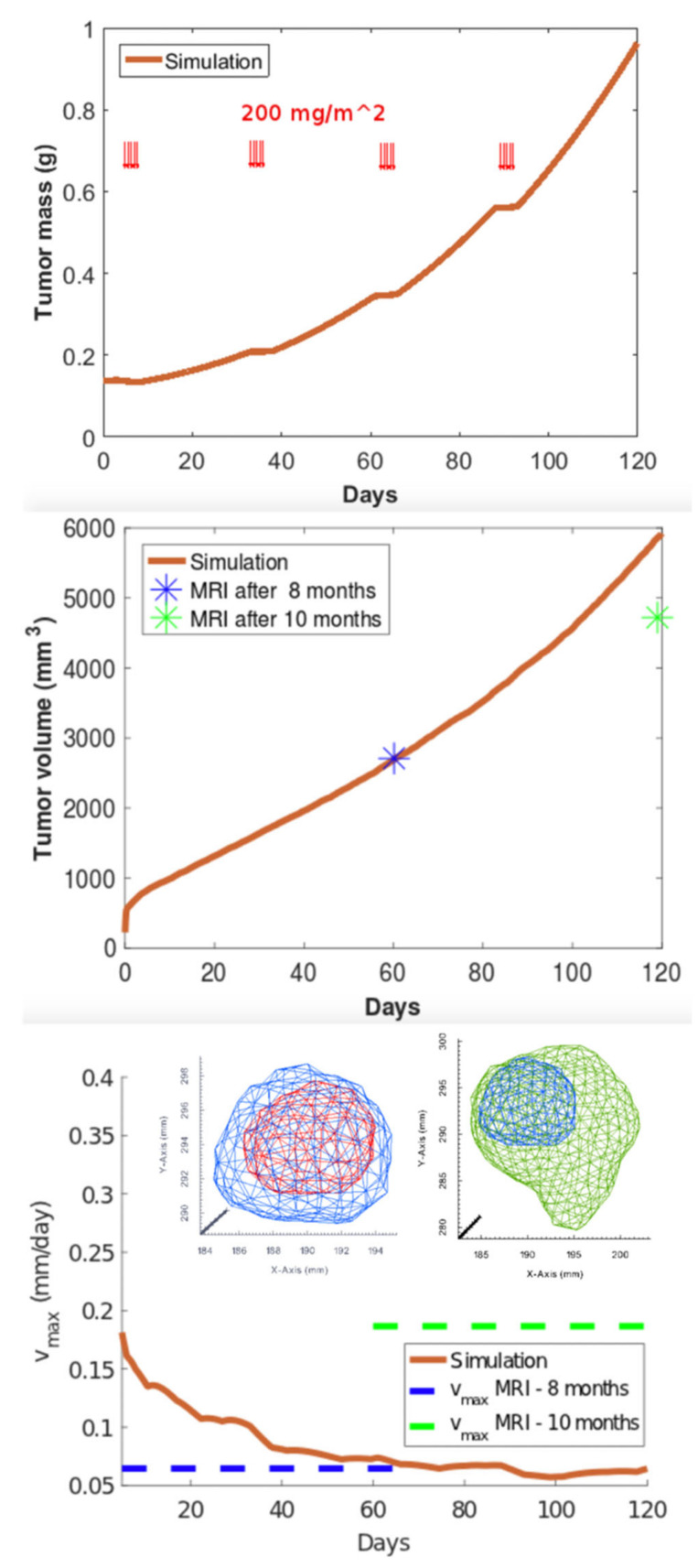
Simulated GBM evolution: mass (**top**), volume (**center**) and spreading velocity (**bottom**). Red arrows in the first figure represent the temporal administration of CT according to Stupp protocol. The insets depict the GBM contours from MRI images (red- month 6; blue-month 8; green-month 10).

We observe that the mass is oscillating during the days of application of the chemo-therapy cycle (Figure 4). The simulated volume matches the value from the data at 8 months, but it fails to match the value at 10 months. In the graph for the expansion velocity from simulation, we have also reported the experimental average velocity from 6 to 8 months and from 8 to 10 months, obtained by calculating the difference of tumor semi-diameters along the coordinate axes of the tumor contours at the different temporal stages (highlighted in blue and green colors), divided by the time period.

## 4. Discussion

GBM is a very complex tumor that includes four molecular subclasses with a wide genotypic and phenotypic variability [67], as highlighted by the 2016 WHO CNS tumors classification [68]. For more than a decade, the Stupp regimen [6] remained the gold standard of GBM treatments, without any improvements in prognosis despite the large amount of pre-clinical and clinical research in this field [13]. Indeed, it is mandatory to look for the application of a personalized model in order to tailor the therapies: early knowledge of the timing and modality of tumor regrowth, in relation to the amount of residual macroscopically tumor volume or microscopically malignant glial cells around the surgical cavity, could guide and modify different clinical decisions.

Many efforts have been aimed to improve the comprehension of the key features of GBM, directing the inception and the development of such a disease. Therefore, during the last decades, the capability of tumor to grow and invade the surrounding tissue has gained the attention of the mathematical and the physical research communities and numerous mathematical models have been proposed in order to analyze different aspects of tumor evolution [27,69].

The knowledge on GBM coming from basic science, preclinical and clinical studies, is continuously being enlarging. In silico neuro-oncology can supply a platform for the integration of all these data in a logical and coherent manner so as to produce clinically relevant models predicting personalized response to available therapeutic options. Mathematical oncology has advanced the idea of targeted plans, regimens and prescription doses. Both analytical and stochastic in silico models simulate and visualize the development, growth and relapse of GBM in real patients. Each modeling approach has its own strengths and weaknesses. Only a direct comparison between models would prove the superiority of one or another. It is also possible that in different frameworks, different models would fit better.

In comparison with the continuum approach, which are considered simpler, keeping predictive significance for a model’s use as a bedside tool, discrete models allow to translate biological processes into model rules more easily, but can be difficult to study analytically and the associated computational cost rapidly increases with the number of modelled cells. This makes it difficult to simulate millimeter or greater sized tumors. Furthermore, while discrete models are capable of describing biophysical processes in significant detail, it may be nontrivial to obtain reliable measurements of model parameters through experiments that can measure the necessary detail at the cell scale [70]. At the same time, the ability of easily incorporating a large amount of data makes stochastic models more useful in research, to compare radiation response to different fractionation regimens, according to patients’ molecular characteristics [65]. All possible treatment scenarios can be evaluated by in silico experiments without considering ethical issues.

Early knowledge of the timing and modality of tumor regrowth, in relation to the amount of surgical excision, could guide and influence some clinical choices. From a surgical point of view, knowing in advance the regions of GBM recurrence could guide the surgeon to extend the surgical removal towards the FLAIR hyperintense envelope where the risk of cancer relapse is higher, achieving thus a supramaximal asportation [11,12]. There is no doubt that a maximal safe resection remains the goal in all cases; in recent years, the concept of “supramaximal” resection, involving resection of both contrast-enhancing tumor and additional resection of the surrounding non-enhancing FLAIR signal, has evolved. As established by Li and coworkers [71] in a large series of patients in 2016, supramaximal resection significantly increases the overall survival if compared to gross total resection. Resection of non-enhancing disease is recommended in cases in which it does not confer additional morbidity [72,73]; our model can be considered as a surgical adjunct to achieve maximal safe surgical resection of the most infiltrated FLAIR hyperintense envelope at a higher risk of tumor recurrence.

As abovementioned, RT is underrepresented in clinical trials. In silico comparison of different radiation regimens can help to fill in the gap created by the paucity of clinical trials on RT in GBM. At the current state of knowledge, the gross target volume includes the area of residual enhancement plus the surgical bed; a margin, typically 2 cm including the hyperintensity in FLAIR, is added to the gross target volume to define the clinical target volume: the clinical target volume remains constant during all the cycle of RT [13,74]. Mathematical neuro-oncology has advanced the idea of tailored plans, regimens and prescription doses. Currently, the standard RT administration according to Stupp protocol contemplates the irradiation of a standardized envelope that is considered health brain with micro-infiltration of malignant cells. Starting from these simulations, it will be possible to elaborate new RT protocol, varying doses or fractioning, and to modify, during the irradiation, the clinical target volume, progressively enlarging it to hit GBM infiltrative cells, since the tumor tends to expand centrifugally in the surrounding healthy parenchyma [22]. Adding radiobiological parameters, such as in more recent stochastic models, could further improve RT planning with a significant benefit for patients.

Regarding CHT [75], knowing in advance the path of tumor regrowth could help the positioning of local delivery tools, such as carmustine wafers or similar devices, reducing complications and better distributing drugs [76]. Indeed, although carmustine wafer implantation had a promising role in the local control of disease as a CHT adjunct [77], this therapeutic approach is associated with higher rates of complications, such as infection, edema, healing defect and seizure [78]. Even local CHT can be targeted and specifically implanted in the region at higher risk of tumor regrowth, with the aim of better disease control minimizing the abovementioned complications. Furthermore, the introduction of adjuvant antiangiogenic factors [79] has been investigated by mathematical modelling, especially by analytical ones. Since the introduction of the grow-or-go multilayer model [37], mathematical neuro-oncology has focused on the association of anti-motility factors with antiangiogenic agents for a more effective treatment of malignant gliomas.

Finally, mathematical modeling has seldom considered the peculiarities of GBM in the elderly population, which represents about half of the GBM population with increasing incidence; their median survival is 6 months regardless of the treatment, which is much shorter of the general GBM population [80]. Recently, a shorter fractionation schemes for the elderly population has been introduced, in association with temozolomide in case of methylated status of MGMT gene promoter [81]. The elderly are a considerable subpopulation of GBM patients and their different response to treatment should be studied in silico.

In order to be clinically useful, mathematical models should continuously incorporate the latest medical advances, from molecular science to imaging techniques. In silico modeling cannot definitely replace clinical trials: real patients’ data are necessary to validate models’ predictions. Nevertheless, it can pave the way to clinical research, saving time and reducing costs. At present, each research group purposes its own model. Yet, the future lies with the integration of modeling approaches, as it is evident with the development of hybrid models, combining continuum and discrete tools [63]. It will be necessary to include the bio-molecular data detectable by routine histopathological analysis in the mathematical model in order to achieve a hybrid discrete-continuous model which could better correlate the different prognostic factors and genetic characteristics to GBM growth simulation [24]. The comparison between different models is an inevitable step towards their clinical application.

Preliminary data derived from the application of our model to a patient-specific case is suggestive in predicting the observed GBM behavior with a high degree of agreement [23]. The first phase of GLIO.MATH study is a pure observational trial in which the first simulations are analyzed using statistical methods for evaluating the model efficiency for clinical use, addressing the accuracy with which the model reproduces clinical data and the degree of confidence with which the model forecasts peculiar quantities of interest in order to continuously refine and calibrate the model with the aim of improving its precision in predicting reliably tumor growth even for long period.

This specific case allows to study model utility in analyzing multifocal tumors and in studying independent evolution of different lesions. We studied the evolution of the posterior temporal mass as a primary tumor which was influenced by systemic CHT. However, malignant glial cells have the capacity to growth and diffuse through white matter tracts where the blood–brain barrier is not significantly damaged and produce a dimensional increase of satellite components. By the use of our model, we are able to study this tumor progression until every lesion maintain a relative independence compared to other localization and especially to eventual surgical cavity where the accumulation of CSF and the alteration of liquor dynamic could modify nutrients diffusion, as it happens between the eighth and tenth month in the analyzed case. These good results could be suggestive for a further application in studying secondary GBM analyzing the progression of lower-grade lesions.

The principal limitation of this model is the lack of consideration of data deriving from molecular biology [82]. Indeed, in our model, biological parameters are estimated from in vivo and in vitro tests on healthy brain tissue and GBM that can be explicitly found in the literature. Our aim is to integrate the purposed model with patient-specific molecular and mechano-biological data as deriving from in vivo assays of surgically removed malignant glial cells. As concerning with future works, in addition to taking in care bio-molecular data, we will focus on refining the study of distribution of blood vessels: in particular, current model considers a homogeneous and permanent distribution of them, thus neglecting the role of angiogenesis in GBM development, which is a hallmark of the disease. Future improvements should consider a patient-specific nutrient supply term, considering both physiologically blood vessels ling mostly in grey matter and dynamic tumor neo-angiogenesis, elaborating data derivable by Perfusion Weighted Imaging techniques on brain perfusion and vessel location and combining them in the model equation with DTI data.

## 5. Conclusions

Mathematical neuro-oncology is an emerging approach to improve GBM comprehension; the presented review has explored the applications and different peculiarities of each model. Emerging data seem to demonstrate that a multidisciplinary approach with the central role of in silico models has the potential to aid clinicians in the customization of therapeutic strategies.

In particular, our model, which takes into account not only biochemical factors such as nutrients availability but also mechanical interactions occurring between the local microenvironment and the tumor, represents one more step toward the definition of a computational tool combining clinical data with a mathematical model, able to capture both chemical and mechanical phenomena driving GBM evolution.

Future efforts will be devoted to the definition of a multiscale approach in order to combine the subcellular and the cellular discrete description into the macroscopic continuous representation of the whole process. Indeed, only a multiscale and multidisciplinary approach combining clinical and radiological data with a mathematical model able to capture phenomena occurring at different scales, has the potential to foster our understanding on GBM evolution in every single patient, in order to tailor specific therapies in the new field of precision medicine and, in particular, of personalized neuro-oncology.

## Figures and Tables

**Table 1:** Principal innovation in the continuum mathematical models of gliomagenesis.

Authors	Key Features	Prediction
Owen LN (1969) [28]	Relation between cell kinetics and growth of the gross tumor	Tumor growth and cell production vs. cell loss
Swanson KR (2002) [29]	Quantification of the spatio-temporal growth and invasion of gliomas in three dimensions	Tumor growth and microscopic invasion
Swanson KR (2000) [5]	Augmented diffusion rates of malignant cells in white matter as compared to grey matter	Pattern of microscopic and submicroscopic invasion of the brain by glioma cells
Jbabdi S (2005) [30]	Implementation of modeled glioma diffusion by means of introduction of brain anisotropy, as detectable with diffusion tensor imaging	Pattern of glioma cells migration
Cristini V (2009) [31]	Role of microenvironment vasculature and chemotaxis in glioma invasive behavior	Pattern of tumor invasiveness
Macklin P (2007) [32]	Implementation the role the properties of microenvironment in detecting cancer morphology	Prediction of tumor 3D morphology and malignant properties
Harpold HLP (2007) [21]	Analyzing the relation between tumor growth velocity and cellular proliferation rate	Survival time
Swanson KR (2008) [20]	Analyzing tumor spreading velocity starting from patient-specific MRI	Survival time
Wang CH (2009) [33]	Quantification of patient-specific kinetic rate of malignant cell proliferation since serial preoperative MRI	Diffusion rate and development of GBM for each patient
Rockne R (2010) [34]	Incorporating the effect of radiation therapy in mathematical model of glioma growth	Tumor dimension after RT protocol
Unkelbach J (2014) [35]	Analysis of malignant cell infiltration by means of FLAIR images and prediction of RT response	Optimization of patient-specific radiation therapy and dosing of fall-off rate
Zhao Y (2015) [36]	Role of angiogenesis in tumor development and aggressiveness	Effect of antiangiogenic drugs
Saut O (2014) [37]	Role of hypoxia in tumor development and invasion	Prediction of tumor behavior (proliferative vs. invasive phenotype)
Colombo MC (2015) [23]	Analyzing patient-specific preoperative DTI in revealing personal heterogeneity and anisotropy of brain tissue	Tumor growth
Lipkova J (2019) [38]	Integration complementary information from MRI and FET-PET to infer tumor cell density in GBM patient to tailor radiotherapy	Individual response to RT
Acerbi F (2021) [24]	Introducing in a continuous mechanical model, the heterogeneity and the anisotropicity of the brain bundles from patient-specific DTI	Tumor growth, invasion and recurrence

**Table 2:** Principal innovation in the discrete and hybrid mathematical models of gliomagenesis.

Authors	Key Features	Prediction
Duchting W (1992) [52]	Development of a 3D spheroid tumor model analyzing cellular cycle phases	Tumor response to different RT fractionation schemes
Wasserman R (1996) [53]	Integrating patient-specific mechanical properties of the tumor, as derived from personal MRI	Tumor growth and neoplastic proliferation
Kansal AR (2000) [54,55]	Detecting tumor behavior using a three-dimensional cellular automaton model	Tumor growth
Dionysiou DD (2004) [56]	Integration in a single four-dimensional simulation model several groups of cells in different phases of the cell cycle	Tumor growth and response to RT
Dionysiou DD (2008) [57]	Incorporation of genetic and molecular factors affecting radiosensitivity	Tumor growth and response to adjuvant therapies
Zheng X (2005) [58]	Analyzing the relation among neovascularization (tumor angiogenesis) and cellular invasiveness using an adaptive, unstructured finite element mesh	Tumor response to RT and antiangiogenic drugs
Frieboes HB (2007) [59]	Combination of analytical and stochastic models linking cellular properties and microenvironment vascularization	Tumor growth
Kim Y (2013) [60]	Analyzing the relation between metabolic stress and biophysical interactions with microenvironment	Experimenting target therapies
Angeli S (2018) [61]	Combination of cellular events which cause tumor proliferation and migration with biomechanical response at tissue level	Tumor infiltration and distant invasion
Gallaher JA (2020) [62]	Combination of MRI data to estimate the role of microenvironment with biopsy data to detect molecular cell properties	Prediction of tumor recurrence and effect of adjuvant therapies

## Data Availability

The data presented in this study are available in the present article. Further data on review search or data regarding clinical cases are available upon request to the corresponding author (F.A.).

## Appendix A. GLIOMATH: Clinical and Radiological Data Collection

Patients older than 18 years old with suspected, newly diagnosed, untreated GBM and eligible for surgical removal or biopsy of their lesion were considered for participating in our trial; exclusion criteria were inability to give consent due to cognitive deficits or language disorders, or, for women, pregnancy or lactation.

The trial was structured into a clinical part, to collect clinical, histological, genetic characteristics of the tumor and radiological findings based on MRI and DTI, and in a subsequent part to develop a multi-scale mathematical model. The last aim was to simulate GBM diffusion starting from the patient-specific data collected after neuroradiological studies.

The patients were enrolled in a prospective observational study; the evaluation was based on the normal clinical practice. The surgical databases and the abovementioned trial have been approved by the Ethical Committee of the Fondazione IRCCS Istituto Neurologico Carlo Besta; written informed consent was obtained for each case.

### Appendix A.1. Preoperative Screening, Radiological Protocol and Surgery

All patients underwent neurological examination, preoperative volumetric MRI including DTI (3 Tesla MRI scan—Philips), and recording of concomitant medications. Patients were scheduled for surgical removal or biopsy as judged by the surgeon; in both cases, the procedures were performed in a standard manner, with any surgical tools, such as neuronavigation, fluorescent visualization by 5-aminolevulinic acid or sodium fluorescein, intraoperative contrast enhanced ultrasound and neurophysiological monitoring, when necessary [83]. The histopathological and molecular analysis of the tumor samples were performed according to the 2016 WHO classification of CNS tumors [68].

Radiological protocol included volumetric axial whole brain T1-weighted MRI at 1 mm × 1 mm × 1 mm spatial resolution and volumetric axial whole brain T1-weighted MRI at same spatial resolution after paramagnetic contrast administration, useful to depict the structural anatomy of the patient’s brain and to calculate the total volume of tumor extension after segmentation procedure; axial whole brain 3D-FLAIR image at 1 mm × 1 mm × 1 mm spatial resolution, useful to delineate the outline of the tumor and peri-tumor rim by suppressing signal from CSF. A set of 147 diffusion-weighted images DTI at 2 mm × 2 mm × 2 mm spatial resolution with anterior–posterior phase encoding direction with different b-value was finally acquired; all diffusion-sensitizing directions were sampled uniformly on the hemisphere and an additional B0 image was acquired with reversed phase encoding direction, as posterior–anterior encoding, for helping in geometric distortion correction.

### Appendix A.2. Postoperative Management

Clinical and radiological post-operative examination were carried out the usual institutional practice. The early clinical evaluation included neurological examination and volumetric contrast-enhanced MRI for estimation of EOR, within 72 h from the surgical intervention. The protocol for early postoperative MRI was the same as performed in preoperative setting without the DTI, that was excluded due to the possibility of artifacts caused by the presence of air in the surgical cavity; the following radiological exams, performed every two months, were performed according to the same protocol of preoperative MRI with DTI.

All patients, upon confirmation of histologic diagnosis of GBM, were offered adjuvant radio- and chemotherapy, according to the Stupp protocol and tailored on the basis of patient age, KPS and methylation status of MGMT gene promoter, according to the EANO guideline [84].

## Appendix B. Mathematical Model

We use a continuous multiphase model of GBM based on the theory of mixtures. We model the tumor as a material made by a phase of glioblastoma cells proliferating into a brain tissue made of CSF, white and grey matter. The GBM phase is described by the volume fraction **ф** = **ф**(***x***,***t***), where ***x*** is the position vector in the physical space and ***t*** is time. The growth of GBM is coupled to the availability of oxygen, whose concentration *n* = *n*(***x***,*t*) obeys the following (diffusion-reaction) differential equation:

(A1) $$\frac{\partial n}{\partial t}=D\left(x,t\right)\;\Delta n+\omega \left(ф,n\right)\;$$

where Δ is the Laplace operator, $D\left(x,t\right)$ is the diffusion matrix that is estimated from the DTI data for each patient and $\omega \left(ф,n\right)$ is the oxygen growth rate, that generally contains a proliferation rate linearly proportional to both φ and n, plus an uptake rate depending on **ф**. The latter term describes the local absorption of chemical energy that feeds the biological proliferation processes driving GBM duplication. The equation describing the evolution of GBM volume fraction is obtained by imposing the Newton’s law of motion for the cellular phase, and it reads:

(A2) $$\frac{\partial }{\partial t}=\nabla \cdot (\;T\left(x,t\right)\;\nabla \mu (ф))+\Omega \left(ф,n\right)$$

where ∇ is the gradient operator, $T\left(x,t\right)$ is a diffusion mobility that is estimated from the DTI data for each patient accounting for the preferential mobility along white matter fiber tracts, μ=μ(ф) is the free energy of the cellular phase and $\Omega \left(ф,n\right)$ is the GBM growth rate, that generally contains a proliferation rate proportional to the uptake rate $\omega \left(ф,n\right)$ of the oxygen, plus an apoptotic rate depending on ф and on the effect of adjuvant therapies. The effect of RT is modelled using a linear-quadratic model for describing the radiation damage, whilst the effect of CHT is given by piecewise constant death rate at the days where the drug is administrated [85]. The free energy is taken as $\mu =f\left(ф\right)-\varepsilon^{2}\Delta$, where $f\left(ф\right)$ in a local interaction potential describing the cell–cell adhesions [86], and ε is the characteristic length of the peri-tumoral front the describes the tumor-host invasion.

Equation (A2) is a fourth order (elliptic) partial differential equation that describes mechanically driven evolution of the diffuse interface model of GBM, that is coupled with the chemically driven evolution of the second order (parabolic) partial differential equation. The model is complemented with no-flux boundary conditions at the skull for the volume fraction of the oxygen concentration. This mathematical approach has notable advantages with respect to sharp-interface models, especially concerning the well-posedness and the numerical implementation [87]. A physiological range of the biological parameters used to model $\Omega \left(ф,n\right)$, $\omega \left(ф,n\right),\;f(ф)$ is finally extracted from previous experimental works [23,24].

Integration of Neuroimaging Data into the Virtual Environment

The MR images are elaborated using the software Slicer3D [88], the toolkit VMTK [89] and the libraries TetGen [90] and VTK [91] to build the computational domain of the virtual brains. The MR images are first smoothed before extracting the brain and tumor boundaries, then we create the computational mesh of tetrahedrons that is refined in the peritumoral area. We also label each element to identify if it is made of white matter, gray matter or CSF, so that each element is associated to the corresponding physical parameters. The raw DTI data were pre-processed at each event performing distortion correction [92], denoising [93] and brain masking [94]. The extraction of the patient-specific data that feed the matrices D and T in the model is performed by matrix inversion using the log-signals. We compared two different models, namely the popular tensor model and the uncontaminated tensor model [95,96]. All images were finally projected into the space of the T1-weighted image of the first event [97].

On the basis of personalized virtual environment, we performed a patient-specific simulation of GBM growth, recurrence and response to therapy. To enforce the well-posedness of the numerical solution, we adapted an iterative algorithm using a variational inequality for projecting the solution into a space of solutions that satisfy a positivity constraint and a separation property for the volume fraction [98,99,100].
